# The Mitochondrial Ca^2+^ Uptake and the Fine-Tuning of Aerobic Metabolism

**DOI:** 10.3389/fphys.2020.554904

**Published:** 2020-10-07

**Authors:** Gaia Gherardi, Halenya Monticelli, Rosario Rizzuto, Cristina Mammucari

**Affiliations:** Department of Biomedical Sciences, University of Padua, Padua, Italy

**Keywords:** mitochondria, aerobic metabolism, mitochondrial calcium uptake, mitochondrial calcium uniporter (MCU), systemic metabolism

## Abstract

Recently, the role of mitochondrial activity in high-energy demand organs and in the orchestration of whole-body metabolism has received renewed attention. In mitochondria, pyruvate oxidation, ensured by efficient mitochondrial pyruvate entry and matrix dehydrogenases activity, generates acetyl CoA that enters the TCA cycle. TCA cycle activity, in turn, provides reducing equivalents and electrons that feed the electron transport chain eventually producing ATP. Mitochondrial Ca^2+^ uptake plays an essential role in the control of aerobic metabolism. Mitochondrial Ca^2+^ accumulation stimulates aerobic metabolism by inducing the activity of three TCA cycle dehydrogenases. In detail, matrix Ca^2+^ indirectly modulates pyruvate dehydrogenase via pyruvate dehydrogenase phosphatase 1, and directly activates isocitrate and α-ketoglutarate dehydrogenases. Here, we will discuss the contribution of mitochondrial Ca^2+^ uptake to the metabolic homeostasis of organs involved in systemic metabolism, including liver, skeletal muscle, and adipose tissue. We will also tackle the role of mitochondrial Ca^2+^ uptake in the heart, a high-energy consuming organ whose function strictly depends on appropriate Ca^2+^ signaling.

## Introduction

Intracellular Ca^2+^ plays a significant role as a second messenger controlling both ubiquitous and tissue-specific processes. The decoy of different triggers and the well-defined spatio-temporal patterns of the Ca^2+^ response to different stimuli warrant the specificity of the cellular response (Berridge, 2001).

The outward translocation of protons across the inner mitochondrial membrane (IMM), determined by the activity of the electron transport chain (ETC), generates a mitochondrial membrane potential (Δψ) that is negative inside. Ca^2+^ influx into energized mitochondria depends on the electrochemical gradient (Deluca and Engstrom, 1961; Vasington and Murphy, 1962) and it is ensured by the activity of the Mitochondrial Calcium Uniporter (MCU), a highly selective channel of the IMM (Kirichok et al., 2004; Baughman et al., 2011; De Stefani et al., 2011). Microdomains of high Ca^2+^ concentration in sites of close proximity between the ER/SR and the mitochondria, the so-called mitochondrial-associated membranes (MAMs), are generated upon Ca^2+^ release from the ER/SR (endoplasmic/sarcoplasmic reticulum) stores, ensuring rapid mitochondrial Ca^2+^ entry (Rizzuto et al., 1993, 1998).

**FIGURE 1:**
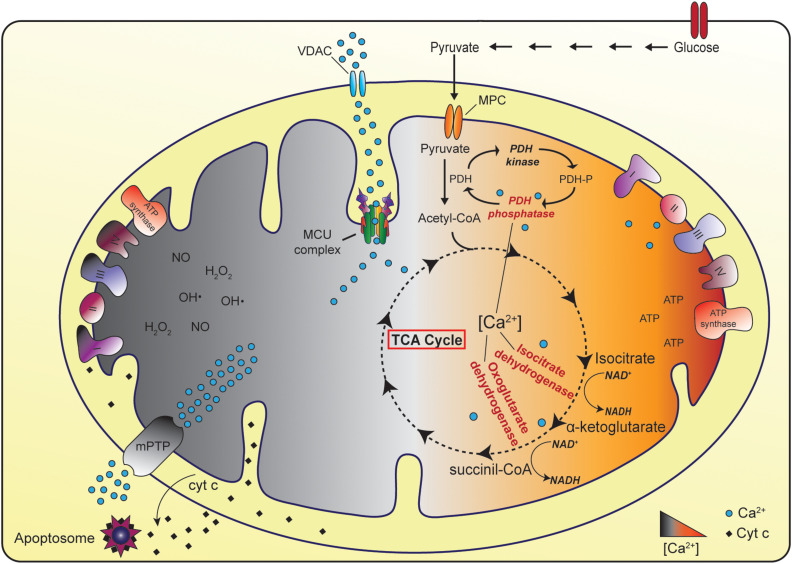
The physiopathological role of mitochondrial Ca^2+^ uptake. In physiological conditions, mitochondrial Ca^2+^ uptake stimulates ATP production thanks to the positive regulation of three Ca^2+^-dependent TCA cycle enzymes (pyruvate dehydrogenase, isocitrate dehydrogenase, and oxoglutarate dehydrogenase). In the cytoplasm glycolytic reactions convert glucose to pyruvate. The Mitochondrial Pyruvate Carrier (MPC) determines the entry of pyruvate into mitochondria, where pyruvate dehydrogenase (PDH) oxidizes pyruvate to acetyl-CoA. Acetyl-CoA enters the TCA cycle leading to the generation of reducing equivalents that feed the electron transport chain and eventually ATP production. Conversely, in pathological settings, mitochondrial Ca^2+^ overload could cause the opening of the mitochondrial Permeability Transition Pore (mPTP) eventually leading to cell death.

In physiological condition, mitochondrial matrix Ca^2+^ regulates the activity of TCA cycle dehydrogenases, thus triggering the generation of reducing equivalents that feed the ETC and eventually ATP production. On the contrary, in pathological settings, mitochondrial Ca^2+^ overload causes the opening of the Permeability Transition Pore (PTP) that results in the rapid collapse of the Δψ and in mitochondrial swelling, together with the release of cytochrome c and pro-apoptotic factors (Figure 1) (Briston et al., 2017; Giorgio et al., 2018).

In this review, we will discuss the role of mitochondrial Ca^2+^ signaling in the control of cell metabolism, with particular emphasis on those organs involved in the regulation of systemic metabolism, including liver, skeletal muscle, and adipose tissue. We will also review the role of mitochondrial Ca^2+^ uptake in the control of heart function, an organ in which this aspect has been widely investigated. We will particularly discuss studies based on the genetic modulation of MCU activity, both in cell lines and in animal models.

## The Molecular Identity and Regulation of the MCU Complex

### The Pore Forming Subunits

To date, three different mitochondrial membrane proteins have been characterized as components of the Ca^2+^ uniporter, i.e., MCU, MCUb, and essential MCU regulator (EMRE).

#### Mitochondrial Calcium Uniporter

All eukaryotes, except for yeasts, display a well-conserved MCU gene (originally known as *CCDC109a*), which encodes a 40 kDa protein composed of two coiled-coil domains and two transmembrane domains, the latter separated by a short but highly conserved loop facing the intermembrane space (IMS). This loop is enriched in acidic residues, essential to confer Ca^2+^ selectivity. MCU was predicted to oligomerize in order to form a functional channel, and this hypothesis was supported by gel separation of native mitochondrial proteins, that highlighted an MCU-containing complex with an apparent molecular weight of 450 kDa (Baughman et al., 2011; De Stefani et al., 2011). Although MCU was predicted to form tetramers by a molecular dynamic approach (Raffaello et al., 2013), the first proposed structure of the *Caenorhabditis elegans* MCU by NMR and cryo-EM techniques envisaged a pentameric assembly (Oxenoid et al., 2016). However, more recently, cryo-EM and/or X-ray structures have been reported for fungal MCU, either alone (Baradaran et al., 2018; Fan et al., 2018; Nguyen et al., 2018; Yoo et al., 2018), or in the presence of EMRE (Wang et al., 2019). These studies demonstrated that fungal MCU complexes are characterized by tetrameric architectures. The tetrameric assembly is conserved also in zebrafish MCU that, by sharing more than 90% homology with human MCU, provides a strong evidence in favor of this architecture in higher eukaryotes (Baradaran et al., 2018).

Mitochondrial calcium uniporter silencing leads to abrogation of mitochondrial Ca^2+^ uptake, while its overexpression triggers both a significant increase of mitochondrial Ca^2+^ transients in intact cells, and an increase in the Ca^2+^ current in mitoplasts (De Stefani et al., 2011; Chaudhuri et al., 2013). Recombinant MCU is sufficient to form a Ca^2+^-selective channel in a planar lipid bilayer *per se* (De Stefani et al., 2011), with a similar current, although not identical, to the one recorded in patch-clamp experiments of isolated mitoplasts (Kirichok et al., 2004). The overall activity of the MCU complex is highly variable among tissues (Fieni et al., 2012). This feature likely integrates different factors: the volume occupied by mitochondria in a specific cell type (Fieni et al., 2012), the relative expression levels of MCU compared to other complex components and/or regulators, including MCUb (Raffaello et al., 2013) and MICU1 (Paillard et al., 2017) and, possibly, the extent of the contact sites between mitochondria and the ER/SR.

#### MCUb

MCUb gene (*CCDC109b*) is conserved in most vertebrates although it is absent in some organisms in which MCU is present (e.g., plants, Nematoda and Arthropoda). MCUb protein, with a molecular weight of 40 kDa, shares 50% sequence homology with MCU and it is similarly organized: it has two coiled-coil domains and two transmembrane domains separated by a loop. A crucial amino acid substitution (E256V) in the loop region neutralizing a negative charge is responsible for the drastic reduction in channel conductivity compared to the MCU (Raffaello et al., 2013). MCUb overexpression decreases mitochondrial Ca^2+^ transients upon agonist stimulation in cells and, when inserted into a planar lipid bilayer, no current is recorded. On the contrary, MCUb silencing triggers a significant increase in mitochondrial Ca^2+^ uptake (Raffaello et al., 2013). This is due to the fact that MCUb forms hetero-oligomers with MCU, negatively affecting the Ca^2+^ permeation through the channel.

Regarding the physiological relevance of MCUb, it should be noted that MCU/MCUb ratio varies greatly among different tissues. For instance, it is higher in the skeletal muscle compared to the heart (Raffaello et al., 2013), in agreement with the different activity of the MCU in different tissues (Fieni et al., 2012).

#### EMRE

EMRE is a 10 kDa protein located in the IMM composed of a transmembrane domain, a short N-terminal domain, and a highly conserved C-terminus enriched in acidic residues (Sancak et al., 2013). Although in a planar lipid bilayer MCU *per se* is sufficient to generate current, experiments performed in EMRE knockout cells demonstrate that this small protein is essential for MCU activity. In cells, EMRE was suggested to modulate the interaction between the pore region and the regulatory subunits of the channel (Sancak et al., 2013). However, in a planar lipid bilayer, MCU and its regulatory subunits MICU1 and MICU2 exhibit the capacity to interact with each other without the presence of EMRE (Patron et al., 2014). Nonetheless, the heterologous reconstitution of human MCU in yeast requires EMRE, indicating that EMRE is needed to assemble a functional channel in metazoan (Kovács-Bogdán et al., 2014). Furthermore, it has been demonstrated that EMRE C-terminus faces the mitochondrial matrix and forms a Ca^2+^-dependent complex with MICU1 and MICU2. Thus, EMRE would act as a sensor for [Ca^2+^] on both sides of the IMM (Vais et al., 2016). However, a different EMRE topology across the IMM has been alternatively suggested, not compatible with the role of EMRE C-terminus as a matrix-Ca^2+^ sensor (Yamamoto et al., 2016). Recently, to determine the relevance of EMRE *in vivo*, an EMRE^–/–^ mouse model was generated (Liu et al., 2020). Studies in these mice demonstrated that EMRE is indeed required for MCU activity, as EMRE^–/–^ isolated mitochondria did not accumulate Ca^2+^. EMRE deletion results in almost complete embryonic lethality in inbred C57Bl6/N mice. However, EMRE^–/–^ animals are viable in a mixed background, although they are smaller and born with less frequency. No differences were detected in terms of basal metabolic functions in the hybrid Bl6/N-CD1 EMRE^–/–^ animals compared to controls. Indeed, neither the total body oxygen consumption nor the body carbon dioxide generation were affected by EMRE deletion. EMRE^–/–^ mice showed normal skeletal muscle function in terms of muscle force and running capacity. In addition, knockout animals displayed regular cardiac function including no changes in IR (ischemia-reperfusion) injury. Thus, similarly to the MCU^–/–^ mouse model generated in a mixed background (Pan et al., 2013; Murphy et al., 2014), adaptations occur to sustain mitochondrial activity in the absence of a functional MCU channel. Finally, Liu et al. (2016) showed that in the absence of MICU1 expression, deletion of one allele of EMRE reduced the mitochondrial Ca^2+^ uptake, suggesting that efforts to modulate the MCU complex could be beneficial for the diseases characterized by Ca^2+^ overload.

### The Regulatory Subunits

**FIGURE 2:**
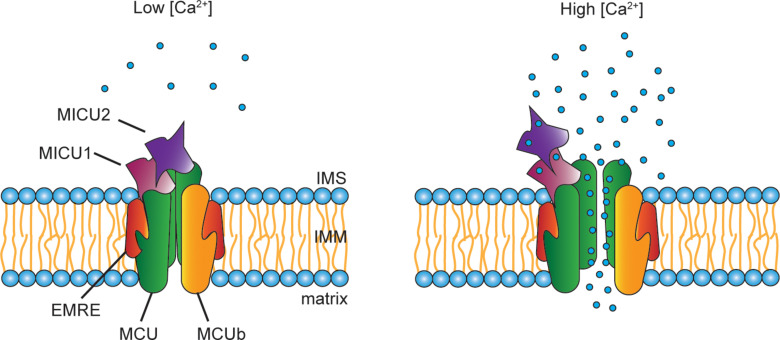
Scheme of the MCU complex. The mitochondrial Ca^2+^ uniporter (MCU) is composed of pore-forming subunits (i.e., MCU, MCUb, and EMRE) and regulatory subunits (i.e., MICU1, MICU2, and MICU3). MCU and the dominant-negative isoform MCUb are assembled into tetrameric complexes that span the IMM. EMRE is the essential MCU regulator, a 10 kDa protein located in the IMM whose absence causes the loss of the uniporter channel activity. A peculiar aspect of the mitochondrial Ca^2+^ uptake is its sigmoidal response to the extra-mitochondrial [Ca^2+^]. This property is due to the presence in the IMS of the MCU regulators, MICU1, MICU2, and, nearly exclusively in the nervous system, MICU3. At low cytosolic [Ca^2+^], little Ca^2+^ enters through the channel. Upon cell stimulation, the increase in cytosolic [Ca^2+^] is sensed by the EF-hands present on both MICU1 and MICU2. Consequently, conformational changes of the regulatory subunits take place, which allows the opening of the channel leading to Ca^2+^ entry inside the mitochondria.

One critical aspect of the mitochondrial Ca^2+^ accumulation is its sigmoidal response to cytoplasmic Ca^2+^ levels. In other words, at low cytosolic [Ca^2+^], mitochondrial Ca^2+^ uptake is negligible, while it exponentially increases when the cytosolic [Ca^2+^] exceeds a certain threshold (Figure 2). The different activity levels are the consequence of a fine-tuned modulation of channel opening, due to the presence of regulatory proteins located in the IMS, which interact with the pore-forming subunits. These regulators belong to the MICU family that comprises MICU1, MICU2 and MICU3, each of them characterized by different expression patterns and specific functions.

#### MICU’s Family of MCU Regulators

MICU1 was the first component of the MCU complex to be identified, and it was described as a critical modulator of mitochondrial Ca^2+^ uptake (Perocchi et al., 2010). MICU1 plays a dual role in the control of MCU activity. On one hand, it keeps the channel closed when extra-mitochondrial Ca^2+^ concentration is below a certain threshold, thus avoiding continuous and sustained mitochondrial Ca^2+^ entry that eventually would cause Ca^2+^ overload. On the other hand, when extra-mitochondrial Ca^2+^ concentration exceeds a certain value, MICU1 acts as a cooperative activator of the MCU, warranting high Ca^2+^ conductivity (Mallilankaraman et al., 2012b; Csordás et al., 2013; Patron et al., 2014). Thus, MICU1 behaves as a gatekeeper of the MCU at low cytosolic Ca^2+^ concentrations and allows efficient mitochondrial Ca^2+^ uptake when cytosolic Ca^2+^ concentration increases (Csordás et al., 2013). Most of MICU1^–/–^ mice die perinatally, and the ones that survive are affected by ataxia and muscle weakness (Antony et al., 2016; Liu et al., 2016).

Later, two MICU1 paralog genes were described: MICU2 (formerly named EFHA1) and MICU3 (formerly named EFHA2), that similarly to MICU1 are located in the IMS (Hung et al., 2014; Lam et al., 2015). While the tissue expression pattern of MICU2 is similar to MICU1, MICU3 is expressed predominantly in the nervous system (Plovanich et al., 2013; Paillard et al., 2017). MICU2 forms an obligate heterodimer with MICU1, stabilized by a disulfide bond through two conserved cysteine residues. In many cell types, the stability of MICU2 depends on the presence of MICU1, as MICU1 knockdown causes a considerable reduction in MICU2 protein levels. This was reported in HeLa cells (Patron et al., 2014) and in patients’ fibroblasts carrying a loss-of-function homozygous deletion of MICU1 (Debattisti et al., 2019). However, in MICU1^–/–^ MEFs as well as in MICU1^–/–^ skeletal muscles, MICU2 protein levels were unaffected (Antony et al., 2016; Debattisti et al., 2019).

MICU1-MICU2 heterodimers regulate the activity of the MCU complex because of the presence of EF-hand domains. Ca^2+^ binding to these domains triggers conformational modifications resulting in channel opening. However, the entity of the Ca^2+^ affinity to the EF-hand domains is still an open issue. Measurements performed by isothermal titration calorimetry indicate that the Kd of the MICU1/Ca^2+^ interaction ranges from 4 to 40 μM (Wang et al., 2014; Vecellio Reane et al., 2016). However, measurements based on the intrinsic tryptophan fluorescence registered a Kd of ∼300 nM (Kamer et al., 2017). Certainly, different methods in diverse experimental conditions could have yielded inhomogeneous results, indicating that this issue needs further clarification. As to the role of MICU2, electrophysiological studies performed in a planar lipid bilayer demonstrated that MICU2 inhibits MCU activity at low cytosolic [Ca^2+^], thus serving as the genuine gatekeeper of the channel (Patron et al., 2014).

Finally, the stoichiometry of the MCU regulators within the complex is still largely unknown. MICU1 multimers undergo molecular rearrangement during Ca^2+^ stimulation (Waldeck-Weiermair et al., 2015). In addition, Paillard et al. (2017) demonstrated that the MICU1/MCU ratio accounts for the different regulatory properties of the MCU complex in the different tissues. For instance, the high MICU1/MCU ratio present in the liver provides high cooperative activation of the channel and, simultaneously, increases the threshold of activation. Accordingly, small changes in cytosolic Ca^2+^ transients in hepatocytes are not sufficient to lead to mitochondrial Ca^2+^ accumulation, which indeed requires sustained cytosolic Ca^2+^ increases. A different scenario occurs in the heart, in which the low MICU1/MCU ratio allows mitochondria to take up Ca^2+^ even at low cytosolic [Ca^2+^]. In this case, the beat-to-beat Ca^2+^ transients are decoded by the MCU with a low level of channel gating and a low grade of cooperativity, thus triggering an integrative Ca^2+^ accumulation (Paillard et al., 2017).

A lot of effort has been made to evaluate the molecular dynamics of the MICU1–MICU2 complex. In particular, Jia and coworkers resolved the human MICU2 structure to investigate the interactions between MICU1 and MICU2 in both the apo and Ca^2+^-bound form. Different residues are involved in the MICU1–MICU2 interaction depending on Ca^2+^ binding to EF-hands motifs. In the apo form, the critical amino acids for the complex formation are Glu242 in MICU1 and Arg352 in MICU2, while in the Ca^2+^-bound state Phe383 of MICU1 interacts with Glu196 in MICU2 (Wu et al., 2019). More recently, the cryo-electron microscopy structures of the human mitochondrial calcium uniporter holocomplex have been resolved in the apo and Ca^2+^-activated states. This study demonstrated that a single MICU1-MICU2 heterodimer is able to gate a channel formed by 4 MCU and 4 EMRE subunits. In agreement with previous reports, the structural analysis confirmed the existence of different conformations according to the [Ca^2+^]. In particular, this study demonstrates that, while at low [Ca^2+^] MICU1 covers the pore, in high [Ca^2+^] MICU1 moves away from the MCU surface to the edge of the MCU–EMRE tetramer, thus allowing pore opening (Fan et al., 2020).

A few years ago, our laboratory characterized an alternative splice variant of MICU1, namely MICU1.1 (Vecellio Reane et al., 2016). MICU1.1 is expressed only in the skeletal muscle, at higher levels compared to MICU1, and in the brain. Compared to MICU1, MICU1.1 contains an additional exon encoding four amino acids (EFWQ). MICU1.1 binds Ca^2+^ more efficiently compared to MICU1, and the heterodimer MICU1.1-MICU2 activates the channel at lower [Ca^2+^] than the MICU1-MICU2 heterodimer. These features are particularly relevant in the skeletal muscle, where MICU1.1 guarantees sustained ATP production. However, how the EFWQ domain affects the Ca^2+^-binding affinity and the protein structure is still under investigation.

MICU3, as already mentioned above, is specifically expressed in the brain (Plovanich et al., 2013). MICU3 forms disulfide bond-mediated dimers with MICU1 but not with MICU2 and acts as an activator of MCU activity (Patron et al., 2019). Accordingly, MICU3 silencing in primary cortical neurons impairs Ca^2+^ signaling suggesting a role in the control neuronal function. Thus, neurons express both MICU1–MICU2 dimers, which guarantee low vicious Ca^2+^ cycling in resting conditions, and MICU1-MICU3 dimers, which decrease the threshold of MCU opening ensuring mitochondrial Ca^2+^ uptake even in the presence of fast and small cytosolic Ca^2+^ transients. Recently, it has been demonstrated that MICU3 is involved in the metabolic flexibility of nerve terminals. MICU3, by tuning the Ca^2+^ sensitivity of the MCU complex, allows presynaptic mitochondria to take up Ca^2+^ in response to small changes in cytosolic [Ca^2+^] and thus to sustain axonal ATP synthesis (Ashrafi et al., 2020).

#### Other Putative MCU Modulators

Other mitochondrial proteins have been identified as putative modulators of MCU channel activity. Briefly, MCUR1, previously known as *CCDC90a*, was described as a modulator of MCU, since its silencing decreases mitochondrial Ca^2+^ accumulation and basal mitochondria matrix [Ca^2+^] in HEK293T cells (Mallilankaraman et al., 2012a). However, Soubridge et al. demonstrated that MCUR1 plays also a critical role in the assembly of complex V, and its silencing drastically decreases the mitochondrial membrane potential (Paupe et al., 2015). Thus, whether MCUR1 is a direct modulator of the MCU, or rather it unspecifically blunts the driving force for mitochondrial Ca^2+^ uptake is still debated. Finally, SLC25A23 is a member of the solute carrier family that transport Mg-ATP/Pi across the IMM (Hoffman et al., 2014). Mutations of SLC25A23 EF-hand domains decrease mitochondrial Ca^2+^ accumulation, thus suggesting that it may play a role in the control of MCU activity.

## Ca^2+^ Targets of the Aerobic Metabolism: the Regulation of Mitochondrial Dehydrogenases

Mitochondrial Ca^2+^ plays various roles impinging on energy metabolism. It contributes to the regulation of shuttle systems through the IMM, that ensure the transport of nucleotides, metabolites, and cofactors (for a review see Rossi et al., 2019). In addition, it has been recently proposed that, within the matrix, Ca^2+^ directly modulates the activity of the ATP synthase (Territo et al., 2000) and the ETC (Glancy et al., 2013).

**FIGURE 3:**
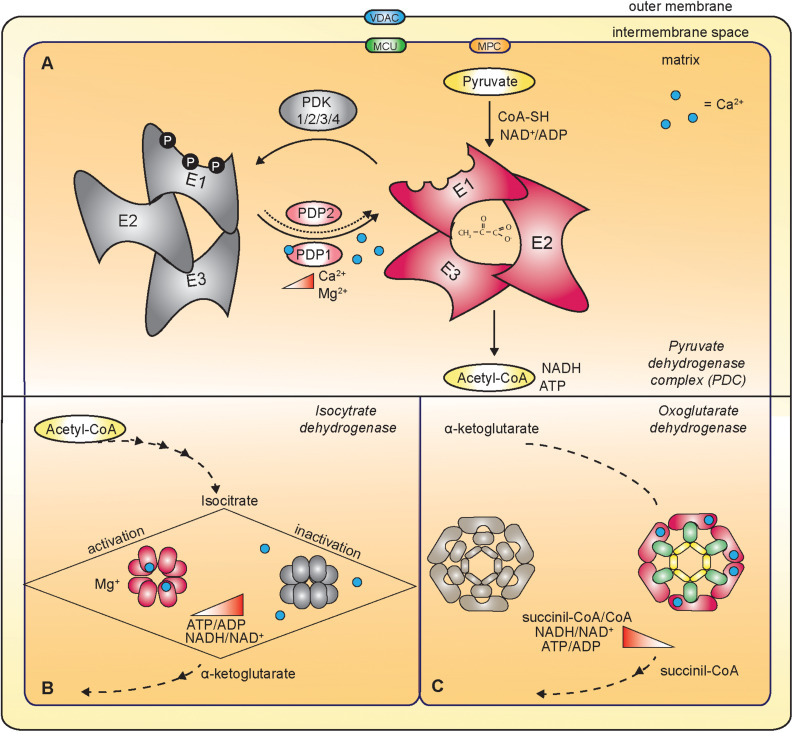
Ca^2+^-dependent regulation of mitochondrial dehydrogenases. **(A)** The mitochondrial Ca^2+^ indirectly regulates the pyruvate dehydrogenase complex (PDC) that oxidizes pyruvate to acetyl-CoA. PDC is composed of multiple copies of three components: pyruvate decarboxylase (E1), dihydrolipoate acetyltransferase (E2) and dihydrolipoate dehydrogenase (E3). In response to increased mitochondrial [Ca^2+^], the Ca^2+^-dependent isoform of pyruvate dehydrogenase phosphatase 1 (PDP1) dephosphorylates E1, thus triggering PDC activation. **(B)** Ca^2+^ directly activates the isocitrate dehydrogenase (IDH). The ATP/ADP ratio negatively regulates the sensitivity of Ca^2+^ binding to IDH. **(C)** Oxoglutarate dehydrogenase (OGDH) is a multienzyme complex similar to the PDC. It is composed of dihydrolipoamide succinyl-transferase subunits (E2, green in the figure), oxoglutarate decarboxylase (E1, pink in the figure) and dihydrolipoamide dehydrogenase (E3, yellow in the figure). Ca^2+^-binding directly regulates the activity of the enzyme.

Most importantly, mitochondrial Ca^2+^ uptake contributes to the regulation of energy production by impinging on the activity of mitochondrial dehydrogenases (Figure 3). In detail, FAD-linked glycerol phosphate dehydrogenase (GPDH) in the IMS, and pyruvate dehydrogenase (PDH), isocitrate dehydrogenase (IDH) and oxoglutarate dehydrogenase (OGDH) in the matrix are directly or indirectly controlled by mitochondrial Ca^2+^ (Denton, 2009).

### FAD-Linked Glycerol Phosphate Dehydrogenase (GPDH)

The glycerol phosphatase shuttle is composed of the mitochondrial FAD-linked glycerol phosphate dehydrogenase and the cytosolic NAD-dependent glycerol phosphate dehydrogenase. This shuttle transfers reducing equivalents from NADH in the cytosol to FADH_2_ in the mitochondrial matrix and eventually to the ETC. FAD-linked GPDH is a transmembrane protein of the IMM and possesses binding sites for glycerol phosphate and Ca^2+^ facing the IMS, thus sensing the cytoplasmic concentration of both these molecules (Cole et al., 1978; Garrib and McMurray, 1986). In particular, two EF-hand domains are responsible for the Ca^2+^-dependent activation of the enzyme (MacDonald and Brown, 1996).

### Pyruvate Dehydrogenase (PDH)

The mammalian pyruvate dehydrogenase complex (PDC) has a molecular weight of about 8 MDa and contains multiple copies of three components that catalyze the conversion of pyruvate to acetyl-CoA. The pyruvate decarboxylase (E1) and the dihydrolipoate dehydrogenase (E3) are attached to a central core formed by the dihydrolipoate acetyltransferase (E2) (Hiromasa et al., 2004). The pyruvate decarboxylase is a tetramer composed of two different subunits (α2 and β2) catalyzing the irreversible step of the reaction. The PDC plays a critical role in the control of cellular metabolism and, accordingly, it is subjected to a fine regulation. Acetyl-CoA and NADH, the end-products of the oxidative reaction, inhibit PDC activity. PDC activity is also inhibited by the reversible phosphorylation of three sites on the E1 subunit (Denton, 2009) catalyzed by pyruvate dehydrogenase kinases (PDKs) and reverted by pyruvate dehydrogenase phosphatases (PDPs). In particular, PDP1 and PDP2 are two isoforms belonging to the 2C/PPM family, with a molecular weight of ∼55 kDa and an Mg^2+^-dependent catalytic subunit (Teague et al., 1982; Karpova et al., 2003). The Ca^2+^-dependent activation of PDP1 results in the dephosphorylation of PDH, and eventually in the conversion of pyruvate to acetyl- CoA (Teague et al., 1982; Turkan et al., 2004). Notably, Ca^2+^ activates the PDP1, but not the PDP2, with a mechanism that is still under investigation. Although the PDP1 sequence contains a putative EF-hand motif (Lawson et al., 1993), the PDP1 crystal structure revealed that this domain is not involved in Ca^2+^ binding (Vassylyev and Symersky, 2007). Further studies, conducted using purified PDP1, showed that the Ca^2+^-binding site might be at the interface between the PDP1 and the E2 with a Kd close to 1 μM (Turkan et al., 2004).

### Isocitrate Dehydrogenase (IDH)

This TCA cycle enzyme catalyzes the decarboxylation of isocitrate to α-ketoglutarate. IDH is an octamer of about 320 KDa and each unit of the octamer is composed of three different subunits (Nichols et al., 1993, 1995). The increase in ATP/ADP and NADH/NAD^+^ ratios inhibits the IDH enzyme, a feature that is shared with the other two Ca^2+^-dependent dehydrogenases, the pyruvate and the α-ketoglutarate dehydrogenases. The sensitivity of Ca^2+^ binding to IDH is controlled by the ATP/ADP ratio. A decrease in the ATP/ADP ratio increases Ca^2+^ binding to IDH, which in turn leads to a decrease of the Km for isocitrate (Rutter and Denton, 1988, 1989).

### Oxoglutarate Dehydrogenase (OGDH)

Oxoglutarate dehydrogenase catalyzes the TCA cycle reaction that converts α-ketoglutarate to succinyl-CoA. Its tetrameric structure shares some similarities with the PDC. It is characterized by a core composed of many subunits of dihydrolipoamide succinyl-transferase (E2), to which 2-oxoglutarate decarboxylase (E1) and dihydrolipoamide dehydrogenase (E3) subunits are attached. Increases in the succinyl-CoA/CoA and NADH/NAD^+^ ratios inhibit OGDH. In contrast to PDC, OGDH is not regulated by phosphorylation events. The Ca^2+^ binding to the OGDH leads to a decrease in the Km for α-ketoglutarate, similar to what happens for the IDH (Denton, 2009).

## The Mitochondrial Ca^2+^ Uptake Regulates Liver Metabolism

Systemic metabolic adaptations to nutritional changes and to alterations in physical activity are necessary for energy homeostasis. In particular, several mechanisms and different organs are involved in the fine-tuned modulation of glucose metabolism. Among them, the liver plays a crucial role because of its capacity to store glucose during the fed state and to release it into circulation during fasting. Several signals control the balance between glycogen storage, glycogenolysis, and gluconeogenesis. Insulin is responsible for glycogen accumulation, whereas hyperglycemic hormones including glucagon and epinephrine are liable for gluconeogenesis and glycogenolysis. Thanks to the seminal work of the Cabbold laboratory, single-cell Ca^2+^ dynamics of rat hepatocytes revealed the oscillatory pattern of cytosolic Ca^2+^ upon hormonal stimulation (Woods et al., 1986, 1987). Subsequently, the spatio-temporal pattern of Ca^2+^ transients was characterized, and the related regulation of Ca^2+^-sensitive proteins and mitochondrial enzymes were deciphered (Hajnóczky et al., 1995; Robb-Gaspers et al., 1998; for a review see Bartlett et al., 2014). In particular, Hajnóczky et al. (1995) demonstrated that the cytosolic Ca^2+^ oscillations translate into mitochondrial Ca^2+^ increases to stimulate aerobic metabolism. In detail, while slow increases of cytosolic Ca^2+^ concentrations are insufficient to activate mitochondrial metabolism, Ca^2+^ release from the ER upon InsP3 receptor opening triggers a rapid cytosolic Ca^2+^ oscillation and consequent mitochondrial Ca^2+^ uptake. Thus, cytosolic [Ca^2+^] increases due to Ca^2+^ leak from stores are distinguished from those due to InsP3 receptors activation, the latter being the ones able to trigger a mitochondrial response. Each mitochondrial [Ca^2+^] spike triggered by specific cytosolic Ca^2+^ oscillation frequencies is accompanied by a maximal activation of the Ca^2+^-sensitive mitochondrial dehydrogenases.

To verify the role of mitochondrial Ca^2+^ uptake in liver metabolism, a hepatocyte-specific MCU^–/–^ mouse model was developed (Tomar et al., 2019). MCU^–/–^ hepatocytes displayed a depletion in mitochondrial matrix Ca^2+^ accompanied by a consequent impairment of the respiratory capacity. Interestingly, in MCU^–/–^ hepatocytes lipid accumulation was observed. Mechanistically, AMPK inactivation was indicated as the trigger of this event. MCU deletion delays cytosolic Ca^2+^ clearance, and this increases Ca^2+^-dependent PP4 phosphatase activity. In turn, PP4 dephosphorylates AMPK. Remarkably, liver-specific AMPK deletion is sufficient to cause hepatic lipid accumulation. In addition, a gain-on-function knock-in MCU mouse model was produced (Tomar et al., 2019). In this model, hepatocytes displayed enhanced mitochondrial Ca^2+^ uptake and respiratory capacity, AMPK was active, and liver triacylglycerol levels were decreased. Of note, MCU-overexpressing hepatocytes were resistant to lipid accumulation when placed in a high-glucose medium.

The regenerative capacity of the liver is of extreme pathophysiological importance and recent evidence demonstrates that Ca^2+^ dynamics takes part in this process. After partial hepatectomy, a cytosolic Ca^2+^ rise promotes the transition of regenerating hepatocytes through the proliferative state allowing liver growth. However, in MICU1^–/–^ liver the cytosolic Ca^2+^ increase triggered by partial hepatectomy causes mitochondrial Ca^2+^ overload and eventually PTP opening. This generates an amplification of the pro-inflammatory phase and a block of the transition through the proliferative phase, leading to an impairment in liver recovery (Antony et al., 2016).

## Mitochondria Ca^2+^ Signaling in the Control of Skeletal Muscle Function

Muscle physiology largely depends on two intracellular organelles: the SR for Ca^2+^ storage and release, and mitochondria for ATP synthesis (Franzini-Armstrong and Jorgensen, 1994). Skeletal muscle requires ATP to drive both actomyosin cross-bridge cycling and cytosolic Ca^2+^ buffering during contraction, the latter being ensured by ATP-dependent SERCA pump activity. Seminal work demonstrated that in rat myotubes mitochondria amplify 4–6 fold the cytosolic [Ca^2+^] increases triggered by various stimuli (Brini et al., 1997). This observation highlighted the pivotal role of mitochondria as essential players in the dynamic regulation of Ca^2+^ signaling in skeletal muscle, in accordance with the reported close apposition of ER and mitochondria that allows efficient Ca^2+^ uptake (Rizzuto et al., 1993, 1998). Electron microscopy studies demonstrated the presence of inter-organelle tethering proteins located at the juxtaposition between SR and mitochondria, that generate a physical coupling between these two organelles (Boncompagni et al., 2009) allowing rapid SR-mitochondria Ca^2+^ transfer. Early studies demonstrated an increase in NADH/NAD^+^ during the transition between resting and working muscle conditions, suggesting that an intracellular Ca^2+^ rise promotes mitochondrial metabolism in skeletal muscle (Sahlin, 1985; Duboc et al., 1988; Kunz, 2001). Recently, it was shown that inhibition of RyR1, thus a decrease in mitochondrial Ca^2+^ uptake, triggers a reduction in the ATP-linked oxygen consumption, pointing out the control of aerobic metabolism by mitochondrial [Ca^2+^] (Díaz-Vegas et al., 2018). Importantly, glycolytic and oxidative muscles differ in terms of their mitochondria volume and metabolic properties. Specifically, slow-twitch fibers rely on oxidative phosphorylation, displaying a great fatigue resistance, while fast-twitch fibers manly rely on glycolysis as a source of ATP.

The importance of mitochondrial Ca^2+^ uptake in skeletal muscle physiology is demonstrated by the disease phenotype of patients carrying mutations in the MICU1 gene. Loss of function mutations cause myopathy, learning difficulties and extrapyramidal movement disorders (Logan et al., 2014). Other patients, carrying a homozygous deletion of the exon 1 of MICU1 show fatigue, lethargy, and weakness (Lewis-Smith et al., 2016). To clarify whether the muscle defects are due to impaired muscle MICU1 function or rather are secondary to neuronal disorders, a skeletal muscle MICU1^–/–^ mouse model was developed (Debattisti et al., 2019). These mice are characterized by muscle weakness, fatigue, and increased susceptibility to damaging contractions, indicating that loss of MICU1 in the muscle is causative of the disease phenotype. In addition, pharmacological inhibition of mitochondrial Ca^2+^ uptake by MICU1 targeting molecules impairs myotubes growth (Di Marco et al., 2020).

MCU deletion in MCU^–/–^ mouse model causes a significant impairment in exercise capacity (Pan et al., 2013). In isolated mitochondria of the MCU^–/–^ muscles resting matrix Ca^2+^ levels are reduced by 75% compared to WT mitochondria, and Ca^2+^-dependent PDH phosphorylation is increased. These data are in accordance with the role of mitochondrial Ca^2+^ accumulation to regulate ATP production necessary to maintain healthy muscle functionality.

Further studies were performed by muscle-restricted modulation of MCU expression (Mammucari et al., 2015). In particular, MCU overexpression and downregulation trigger muscle hypertrophy and atrophy, respectively. The control of skeletal muscle mass by mitochondrial Ca^2+^ modulation is due to the activation of two major hypertrophic pathways of skeletal muscle, PGC-1α4 and IGF1-AKT/PKB. Surprisingly, these effects are independent of the control of aerobic metabolism, as demonstrated by various pieces of evidence. Firstly, PDH activity, although defective in MCU silenced muscles, was unaffected in MCU overexpressing muscles and, secondly, hypertrophy was comparable in both oxidative and glycolytic muscles (Mammucari et al., 2015).

A muscle-specific MCU knockout mouse (skMCU^–/–^), besides corroborating the role of MCU in maintaining muscle mass, helped to elucidate the contribution of mitochondrial Ca^2+^ uptake in muscle metabolism. Furthermore, this animal model was informative on how the MCU-dependent skeletal muscle oxidative metabolism systemically impinges on substrate availability (Gherardi et al., 2018). Metabolically, skMCU^–/–^ mice were characterized by defective glucose oxidation, which was evident by increased blood lactate and by decreased oxygen consumption rate (OCR) of myofibers kept in a high-glucose medium. Skeletal muscle glucose uptake was increased as well as glycogen content, indicative of unaffected insulin sensitivity but inefficient glucose utilization.

Despite reduced muscle force and running capacity during exhaustion exercise, muscle performance was not dramatically impaired. This was due to an increase in fatty acids (FA) oxidation, which significantly contributed to basal OCR (Gherardi et al., 2018). A second skeletal muscle-specific MCU KO model was characterized on the one hand by reduced sprinting exercise performance, on the other hand by enhanced muscle performance under condition of fatigue, where the FA metabolism is predominant (Kwong et al., 2018). Because of the altered muscle glucose utilization, liver and adipose tissue responded with enhanced catabolism. Hepatic gluconeogenesis, lipolysis and thus circulating ketone bodies were increased. Furthermore, adipose tissue lipolysis was enhanced, resulting in a reduction of the body fat mass both in adulthood (Gherardi et al., 2018) and in aging (Kwong et al., 2018). The main trigger of this metabolic rewiring is the reduced pyruvate dehydrogenase activity, which is tightly controlled by mitochondrial Ca^2+^ as indicated by genetic experiments (Gherardi et al., 2018).

In accordance, a skeletal muscle-specific mitochondrial pyruvate carrier (MPC) knockout mouse strikingly resembled the phenotype of skMCU^–/–^ mouse. In skeletal muscle, MPC deletion decreased glucose oxidation despite increased glucose uptake and lactate production. This triggered enhanced FA oxidation and Cori cycling, with an overall increase in energy expenditure. Thus, skeletal-muscle restricted MPC deletion accelerated fat mass loss when mice were fed again with a chow diet after high-fat diet-induced obesity (Sharma et al., 2019). Altogether these studies support a model in which impaired mitochondrial Ca^2+^ uptake and mitochondrial pyruvate entry inhibition lead to similar defects in glucose oxidation, which result in overlapping local and systemic metabolic adaptations. These data point to impaired pyruvate oxidation as the main contributor to the observed metabolic rewiring.

## Cardiac Energetics Is Essential for Proper Heart Function

In the last decade the contribution of mitochondrial Ca^2+^ uptake to the regulation of cardiac energetics has been deeply investigated. Pozzan and coworkers demonstrated that mitochondrial Ca^2+^ transients occur beat-to-beat, and participate in shaping the systolic Ca^2+^ peaks in neonatal cardiomyocytes. The presence of high [Ca^2+^] microdomains generated at the SR/mitochondria contacts allows mitochondrial Ca^2+^ uptake during systole resulting in a significant buffering of cytosolic Ca^2+^ peaks. Ca^2+^ is eventually released back to the cytoplasm during diastole because of the presence of the Na^2+^/Ca^2+^ exchanger and of the Ca^2+^/H^+^ antiporter. In addition, the genetic modulation of MCU, thus the alteration of mitochondrial Ca^2+^ accumulation, directly affects the amplitude of the cytoplasmic Ca^2+^ oscillations in neonatal cardiomyocytes (Drago et al., 2012). However, whether this scenario occurs in adult cardiomyocytes and in the heart, in which it was proposed that mitochondrial Ca^2+^ entry does not rely on a cytosolic Ca^2+^ threshold, is still a matter of debate (Boyman et al., 2014).

In the last few years, many *in vivo* mouse models have been generated to investigate the role of mitochondrial Ca^2+^ uptake in the control of heart function. According to both constitutive and cardiac-specific MCU^–/–^ mouse models, MCU is dispensable for mitochondria bioenergetics in the heart at baseline (Pan et al., 2013; Kwong et al., 2015). The heart function of the constitutive MCU knockout mouse is unaffected upon β-adrenergic stimulation, suggesting that long-term adaptations may occur (Holmström et al., 2015). However, in the inducible cardiac-specific MCU knockout mouse, during the fight or flight response an increase in the mitochondrial ATP production is required to support the elevated energy demand of the augmented heart rate (Kwong et al., 2015; Luongo et al., 2015). In addition, hearts expressing a dominant-negative MCU or overexpressing the MCUb treated with the β-adrenergic agonist isoproterenol show an impairment in the heart rate acceleration (Wu et al., 2015; Lambert et al., 2019). In the conditional MCUb heart-specific transgenic mouse model mitochondrial bioenergetics was impaired because of increased PDH phosphorylation levels leading to a decrease in the maximal respiration and in the reserve capacity. The impairment in the heart responsiveness was highlighted by the fact that these mice did not survive after IR injury. However, long-term induction of MCUb expression was accompanied by a rescue of the cardiac function and reduced infarct size, most likely because of compensatory mechanisms triggering PDH dephosphorylation and thus activation (Lambert et al., 2019). In addition, a change in the stoichiometry of the MCU complex was observed in the hearts of these mice. MCU, MICU1, and MICU2 levels were increased despite decreased association of MICU1 and MICU2 to the MCU complex (Lambert et al., 2019).

The inducible cardiac-specific MCU^–/–^ mouse model was further investigated to assess *ex vivo* cardiac functions (Altamimi et al., 2019). Surprisingly, the isolated MCU^–/–^ hearts showed enhanced cardiac function even at baseline, which persisted after isoproterenol stimulation. Thus, heart-specific mitochondrial Ca^2+^ uptake inhibition hampers the cardiac response to β-adrenergic stimulation *in vivo* (Kwong et al., 2015; Luongo et al., 2015; Wu et al., 2015), but not *ex vivo* (Altamimi et al., 2019). This suggests that the whole-body adrenergic response has a different impact on heart function compared to direct stimulation. A detailed analysis of energetic substrates preference demonstrated that the MCU-deleted hearts rely on increased FA oxidation in response to β-adrenergic stimulation. FA oxidation correlates with a decrease in malonyl-CoA levels, and with increased acetylation of the β-oxidation enzyme β-hydroxyacyl-CoA dehydrogenase (β-HAD) (Altamimi et al., 2019).

The main player of mitochondrial Ca^2+^ efflux, essential for the maintenance of mitochondrial Ca^2+^ homeostasis, is the mitochondrial Na^+^/Ca^2+^ exchanger (NCLX), which is encoded by the Slc8b1 gene (Palty et al., 2010). Recent work helped to elucidate the consequence of Ca^2+^ efflux impairment on heart function. The conditional cardiomyocyte-specific Slc8b1 deletion causes sudden death in most animals due to acute myocardial dysfunction and fulminant heart failure (Luongo et al., 2017). Slc8b1 knockout hearts display sarcomere disorganization, necrotic cell death and fibrosis, accompanied by left ventricle dilation and decreased function. Mechanistically, activation of the mPTP was responsible for the phenotype, as demonstrated by the fact that mating of these mice with cyclophilin D (CypD)-null mice rescues heart function and survival. On the contrary, cardiac-specific NCLX transgenic hearts were characterized by increased efflux capacity, decreased mPTP activation, and were protected against IR injury and ischemic heart failure.

Also, perturbations in the pyruvate dehydrogenase activity in the heart cause metabolic dysfunction and subsequently, cardiomyopathy. Patel and coworkers generated a heart and skeletal muscle-specific PDH knockout mouse (H/MS-PDCKO) (Sidhu et al., 2008). PDC activity is absent in both skeletal muscle and heart of these animals. All the H/MS-PDCKO mice died 7 days post-weaning on a chow diet, and the death was due to an impairment of the left ventricular systolic function, demonstrating that the consequent increase in the glycolysis rate was not sufficient to produce the ATP required for normal cardiac function. In contrast, when H/MS-PDCKO mice were weaned on a high-fat diet they survived for many months. However, even in this case, these mice developed left ventricular hypertrophy when they returned to a chow diet. These data suggest that PDC activity ensures metabolic flexibility in the hearts. Furthermore, Gopal et al. (2018) generated an inducible cardiac-specific PDH knockout model. In these mice, substrate utilization and cardiac function were monitored. The authors observed a metabolic rewiring which includes a reduction in the myocardial glucose oxidation and an increase in the FA oxidation. This metabolic switch did not alter the systolic function, however it impaired the diastolic function causing a cardiomyopathy-like phenotype similar to diabetes or obesity.

These data suggest that, like in skeletal muscle, decreased mitochondrial Ca^2+^ uptake and impaired pyruvate oxidation increase β-oxidation in the heart. Recent work by Wescott et al. (2019) indicates that while mitochondrial [Ca^2+^] regulates the entry of pyruvate and glutamate into the TCA cycle, lipid oxidation is not controlled by mitochondrial [Ca^2+^]. Thus, in a context of mitochondrial Ca^2+^ impairment, fatty acids are readily oxidized to sustain energy demand.

## Ca^2+^ Signaling Plays a Central Role in Adipose Tissue Homeostasis

The study of the role of Ca^2+^ homeostasis in adipose tissue biology represents a relatively new area of research. Different pieces of evidence suggest that Ca^2+^ dysregulation may affect adipocyte function. Accordingly, it has been demonstrated that intracellular Ca^2+^ concentration regulates adipocyte lipid metabolism, thus controlling adipogenesis. Shi et al. (2000) showed that cytosolic [Ca^2+^] plays a biphasic regulatory role in adipocyte differentiation: on the one hand, an increase in cytosolic [Ca^2+^] suppresses the early stages of murine adipogenesis; on the other hand, it promotes the late stages of adipose differentiation. Most importantly, elevated intracellular [Ca^2+^] in adipocyte appears to contribute to the insulin-resistance of elderly hypertensive obese patients. Strikingly, 1 month of therapy with nitrendipine, a Ca^2+^ channel blocker, reduced blood pressure, decreased plasma insulin upon glucose stimulation to control values, and restored normal adipocyte glucose uptake (Byyny et al., 1992).

Specifically in the brown adipose tissue (BAT), early work on isolated adipocytes demonstrated that norepinephrine stimulation mobilizes intracellular Ca^2+^ stores (Connoly et al., 1984), and this was later confirmed by subsequent studies (Lee et al., 1993; Leaver and Pappone, 2002; Nakagaki et al., 2005; Chen et al., 2017). However, the role of Ca^2+^ signaling in BAT thermogenesis remained largely unexplored until recently, when Kcnk3, a K^+^ channel present on the brown adipocyte plasma membrane, was identified as a negative regulator of thermogenesis (Chen et al., 2017). In detail, cytosolic Ca^2+^ levels upon adrenergic stimulation are increased in Kcnk3-deficient brown adipocytes, suggesting that Kcnk3 limits NE-induced cytosolic [Ca^2+^] influx, which results in decreased cAMP production, thus attenuating lipolysis and thermogenic respiration.

It is well established that oxidative metabolism and, in general, mitochondrial bioenergetics are altered in metabolic syndrome, obesity and type 2 diabetes (Heinonen et al., 2019), and that normalizing mitochondrial function has the potential to restore insulin sensitivity. As an example, the treatment with rosiglitazone, a PPARγ agonist and a firmly established drug for type 2 diabetes (Lebovitz, 2019), triggers increased mitochondrial respiration and FA oxidation in ob/ob mice adipocytes (Wilson-Fritch et al., 2004) and induces the expression of mitochondrial structural proteins and cellular antioxidant enzymes (Rong et al., 2007). Nonetheless, while the contribution of mitochondrial metabolism to adipose tissue homeostasis is well known, the role of mitochondrial Ca^2+^ signaling is still largely unexplored. Recently, the link between MCU activity and the development of obesity and diabetes was investigated (Wright et al., 2017). In 3T3-L1 adipocytes, insulin resistance was accompanied by an increase of MCU and MICU1 expression and mitochondrial Ca^2+^ uptake. Accordingly, the expression of MCU complex components was increased in the visceral adipose tissue (VAT) of obese patients, both pre-diabetic and diabetic, compared to lean individuals. Interestingly, upon bariatric surgery, the expression of MCU complex components in patients’ VAT returned to physiological levels, suggesting that mitochondrial Ca^2+^ accumulation plays an active role in this pathology. Mcub overexpression, although insufficient to improve the defect in insulin-stimulated glucose uptake of insulin-resistant cells, restored ROS production to normal levels. In addition, Mcub overexpression reduced TNF-alpha and IL-6 expression, suggesting a possible role of mitochondrial Ca^2+^ uptake in obesity-associated inflammation (Wright et al., 2017). Recently, a BAT-specific MCU knockout mouse was characterized (Flicker et al., 2019). Despite the complete lack of mitochondrial Ca^2+^ uptake, no effects were observed in terms of cold tolerance and diet-induced obesity, suggesting that MCU is dispensable for BAT thermogenesis and metabolism. These last two studies reveal that MCU, in adipose tissue, plays different roles in pathology versus physiology. However, further work is needed to clarify the different role of mitochondrial Ca^2+^ accumulation in metabolically different adipose tissue compartments, both in health and in disease states.

Further insights into the role of Ca^2+^ signaling in the regulation of adipose tissue metabolism comes from studies conducted in *Drosophila melanogaster*. In this animal model the fat body, which is considered the equivalent of the mammalian liver, adipose tissue, hematopoietic and immune systems, senses nutrient availability and controls the metabolic responses (Hotamisligil, 2006). Concerning the Ca^2+^ signaling, Stim, an ER membrane protein involved in store-operated Ca^2+^ entry (SOCE), was identified by RNAi screen among genes affecting adiposity in the fly (Baumbach et al., 2014). Stim downregulation in fat storage tissue decreases cytosolic Ca^2+^ levels and leads to an increase in the overall adiposity. This is due to the remote activation of the orexigenic short neuropeptide F in the brain (sNPF). Along with hyperphagia, the Stim-sNPF axis controls lipid metabolism, resulting in adipose tissue hypertrophy. Further evidence on the role of Ca^2+^ homeostasis in *D. melanogaster* lipid metabolism comes from studies on InsP3 receptor mutants, which are obese and display altered FA utilization (Subramanian et al., 2013). In addition, Huang and coworkers showed that dSeipin, which plays a central role in the lipid metabolism, interacts with dSERCA modulating the cytosolic [Ca^2+^] (Bi et al., 2014). Mutations in dSeipin cause a reduction in SERCA activity and a consequent decrease in ER Ca^2+^ levels, leading to an impairment in the fat storage. Moreover, in dSeipin mutants, mitochondrial Ca^2+^ uptake is reduced, triggering a decrease in TCA cycle activity and accumulation of citrate, a key component of lipogenesis. The lipid storage defect in dSeipin mutant fat cells is rescued by the recovery of mitochondrial [Ca^2+^] or by the restoration of normal citrate levels (Ding et al., 2018).

## Concluding Remarks

About 10 years after the identification of the genes encoding a fundamental MCU regulator (i.e., MICU1, Perocchi et al., 2010) and MCU itself (Baughman et al., 2011; De Stefani et al., 2011), a bulk of information on the channel function and the consequences of its dysfunction has been collected. Most of the studies are based on genetic manipulation of the MCU complex components in specific subcellular compartments or on the disease phenotype of patients carrying mutations in MCU-associated genes. Recently, studies conducted in metabolically active tissues have highlighted the importance of the MCU to warrant metabolic flexibility in the utilization of oxidable substrates. Lack of Ca^2+^ entry causes defective oxidative metabolism in liver, heart, skeletal muscle and adipose tissue, and rewiring of substrate utilization that determines increased fatty acids oxidation in the heart and skeletal muscle, and lipid accumulation in the liver. Future work will further shed light on the importance of mitochondrial Ca^2+^ uptake and pyruvate oxidation on critical issues of metabolism, and on the cross-talk between different organs.

## Author Contributions

GG and CM wrote the manuscript. HM prepared the figures. RR contributed to the critical insights. All authors contributed to the article and approved the submitted version.

## Conflict of Interest

The authors declare that the research was conducted in the absence of any commercial or financial relationships that could be construed as a potential conflict of interest.
